# Multidrug resistant bacterial infections in severely ill COVID-19 patients admitted in a national referral and teaching hospital, Kenya

**DOI:** 10.1186/s12879-022-07885-3

**Published:** 2022-11-22

**Authors:** Jeniffer Munyiva Mutua, John Mwaniki Njeru, Abednego Moki Musyoki

**Affiliations:** 1 Department of Laboratory Medicine, Kenyatta National Hospital, P.O. Box 20723-00202, Nairobi, Kenya; 2 Centre for Medical Microbiology, Kenya Medical Research Institute, P.O. Box 19464-00200, Nairobi, Kenya; 3 Department of Medical Laboratory Sciences, Kenyatta University, P.O. BOX 43844-00100, Nairobi, Kenya

**Keywords:** COVID-19, SARS-CoV-2, Bacterial infections, Multidrug resistant, Severely ill patients

## Abstract

**Background:**

Bacterial infections are a common complication in patients with seasonal viral respiratory tract infections and are associated with poor prognosis, increased risk of intensive care unit admission and 29–55% mortality. Yet, there is limited data on the burden of bacterial infections among COVID-19 patients in Africa, where underdeveloped healthcare systems are likely to play a pertinent role in the epidemiology of the COVID-19 pandemic. Here, we evaluated the etiologies, antimicrobial resistance profiles, risk factors, and outcomes of bacterial infections in severely ill COVID-19 patients.

**Methods:**

A descriptive cross-sectional study design was adopted in severely ill COVID-19 patients at Kenyatta National Hospital, Kenya, from October to December 2021. We used a structured questionnaire and case report forms to collect sociodemographics, clinical presentation, and hospitalization outcome data. Blood, nasal/oropharyngeal swabs and tracheal aspirate samples were collected based on the patient's clinical presentation and transported to the Kenyatta National Hospital microbiology laboratory for immediate processing following the standard bacteriological procedures.

**Results:**

We found at least one bacterial infection in 44.2% (53/120) of the patients sampled, with a 31.7% mortality rate. Pathogens were mainly from the upper respiratory tract (62.7%, 42/67), with gram-negative bacteria dominating (73.1%, 49/67). Males were about three times more likely to acquire bacterial infection (*p* = 0.015). Those aged 25 to 44 years (p = 0.009), immunized against SARS-CoV-2 (p = 0.027), and admitted to the infectious disease unit ward (p = 0.031) for a short length of stay (0–5 days, p < 0.001) were more likely to have a positive outcome. Multidrug-resistant isolates were the majority (64.3%, 46/67), mainly gram-negative bacteria (69.6%, 32/46). The predominant multidrug-resistant phenotypes were in *Enterococcus cloacae* (42.9%, 3/7), *Klebsiella pneumonia* (25%, 4/16), and *Escherichia coli* (40%, 2/5).

**Conclusion:**

Our findings highlight a high prevalence of multidrug-resistant bacterial infections in severely ill COVID-19 patients, with male gender as a risk factor for bacterial infection. Elderly Patients, non-SARS-CoV-2 vaccination, intensive care unit admission, and long length of hospital stay were associated with poor outcomes. There is a need to emphasize strict adherence to infection and prevention at KNH-IDU and antimicrobial stewardship in line with local and global AMR control action plans.

## Background

Coronavirus Disease-2019 (COVID-19), which is caused by severe acute respiratory syndrome Coronavirus 2 (SARS-CoV-2), is a febrile respiratory illness that may progress to pneumonia and respiratory failure and poses a global public health challenge [1]. Over 545 million infections and over 6.3 million deaths [2] had occurred by the time we wrote this publication, and the mortality rates are disproportionately higher in elderly patients [3] than in other age groups. Published literature shows that secondary bacterial coinfections increase the severity of viral respiratory infections [4] and significantly contribute to increased morbidity and mortality [5–7].

As reported in influenza, viral-induced epithelial immune damage and immune downregulation favor bacterial infections or colonization [8–11]; however, the role of bacterial infections in the pathogenesis of SARS-CoV-2 is not well understood. Some literature suggests that bacterial infections in COVID-19 are a minority, with prevalence ranging from 0 to 6.9%, and require no antimicrobial prescription [6, 12, 13]; but other findings, especially from Asia show a significantly higher burden, with up to 95.65% prevalence [14] and 50–83% mortality [15, 16]. Community-onset of bacterial infections in COVID-19 is low [17], but most bacterial infections occur after hospital admission, especially in the intensive care unit (ICU) [18, 19].

According to Shafran et al.*, *bacterial infections are a common complication associated with worse outcomes in COVID-19 patients than influenza patients, and careful surveillance and prompt antibiotic treatment may benefit selected patients [20]. Yet, there is limited data on the burden of bacterial infections among COVID-19 patients in Africa, where poor sanitation, inadequate potable water, and underdeveloped healthcare systems [21, 22] are likely to play a pertinent role in the epidemiology of the COVID-19 pandemic. Additionally, the prevalence of bacterial infections and microbiological etiologies in critically ill COVID-19 patients in many developing countries is poorly understood. Due to the frequent use of invasive devices, hospitalized critically ill COVID-19 patients are at high risk of nosocomial infections, mostly bacteremia and respiratory tract infections, within 10–15 days of admission [23, 24].

Differentiating COVID-19 from bacterial pneumonia is difficult. As a result, COVID-19 patients are frequently prescribed broad-spectrum antibiotics, without laboratory-based evidence, as part of clinical care to treat and prevent bacterial infections [25, 26]. For instance, 95% of 191 COVID-19 patients were empirically treated with antibiotics in Wuhan [14, 26]. Elsewhere, in a randomly sampled cohort of 1705 patients hospitalized with COVID-19 in 38 Michigan hospitals, 56.6% (27–84%) of the patients were prescribed empiric antibacterial therapy at admission despite low (3.5%) community-onset of bacterial infections [17]. The indiscriminate use of antibiotics during the COVID-19 pandemic is likely to exacerbate the global antimicrobial resistance (AMR) menace, with profound implications for global health and the economy [27–29]. A high prevalence of carbapenem-resistant bacterial infections in COVID-19 patients admitted to ICUs was reported in Iran [15]. With an estimated 10 million deaths and 100 billion dollars in economic loss annually due to MDR bacteria by 2050 [21–23], a better understanding of the local epidemiology of bacterial infections in COVID-19 can inform judicious antimicrobials use in line with the national policy and global action plan for prevention and containment of AMR [30].

Previous studies show that gram-negative bacteria are the predominant cause of infections in COVID-19 patients [13–15, 31], but most of these reports are from outside Africa, and bacterial etiologies and antimicrobial resistance are subject to geographical variation. It is imperative to evaluate the etiologies and AMR of bacterial infections in COVID-19 patients to inform policymakers on local empiric therapy design and prevention interventions that can mitigate AMR spread in line with national and global strategies. In this study, we evaluated the etiologies, AMR profiles, risk factors, and outcomes of bacterial infections in critically ill COVID-19 patients admitted to ICU in a single tertiary national teaching and referral hospital in Kenya.

## Methods

### Study area and study design, data collection, and outcomes

This study was done at Kenyatta National Hospital (KNH) at the Infectious Disease Unit (KNH-IDU), Kenya. A descriptive cross-sectional study design was conducted between October and December 2021. Through a purposive sampling technique, 120- Real Time reverse transcription and quantitative Polymerase Chain Reaction (RT-qPCR) confirmed severely ill COVID-19 patients were recruited. Patients’ legal representatives or their guardians who declined to give consent for their patients’ participation in this study were excluded.

A structured questionnaire and case report forms were used to collect data on sociodemographics, and clinical presentation, and hospitalization outcomes. Based on clinical presentation of the patient and the decision of the treating physician, blood, swabs (nasopharyngeal & oropharyngeal) and tracheal aspirate samples were collected following standard recommended procedures. Thereafter, samples were transported to the KNH microbiology laboratory in a cool box and processed immediately. Bacteriological isolation was done following the standard bacteriological procedures (CLSI, 2021) [32]. Briefly, nasopharyngeal (NP) swabs, oropharyngeal (OP) swabs and tracheal aspirates were streaked onto sheep blood agar (Oxoid, United Kingdom) and MacConkey (Oxoid, United Kingdom) and incubated at 37 °C overnight. Blood samples were collected directly into the sterile commercial blood culture broth and loaded to the BACT/ALERT® VIRTUO 3D Microbial Detection Systems (bioMérieux, Marcy l'Etoile, France). Samples flagging positive were sub-cultured onto chocolate blood agar (CBA), (Oxoid, United Kingdom), MacConkey (Oxoid, United Kingdom) and sheep blood agar (Oxoid, United Kingdom) and incubated at 37 °C overnight at both ambient air and 5% CO_2_. Isolates were identified using VITEK Mass Spectrometry System Matrix Assisted Laser Desorption Ionization Time- of- Fight (VITEK MS MALDI-TOF) (BioMérieux, Marcy l'Etoile, France). *Escherichia coli* ATCC 8739 was used as a Quality Control (QC) organism.

Antimicrobial susceptibility testing (AST) was done using the VITEK 2 COMPACT system (bioMérieux, Marcy l'Etoile, France*)* in accordance with the CLSI (2021) guidelines [32]. We used AST GP 580 and AST GP 586 cards for sensitivity testing of gram-positive bacteria (GPB), with *Enterococcus feacalis* (ATCC 29212) and *Staphylococcus aureus* (ATCC 29213) as quality control (QC) organisms. The antibiotic panels tested were: benzylpenicillin (BP), levofloxacin (LVX), erythromycin (ERY), linezolid (LZD), teicoplanin (TEC), vancomycin (VAN), tetracycline (TET), and tigecycline (TGC). Using AST GN 83 card, and *Pseudomonas aeruginosa* (ATCC 27853) and *Escherichia coli* (ATCC 25922) as QC organisms, GNB were tested for susceptibility to the following antibiotic panels: amoxicillin/clavulanic acid (AMC), ampicillin/sulbactam (SAM), piperacillin/tazobactam (TZP), cefotaxime (CTX), ceftazidime (CAZ), ceftriaxone (CRO), cefepime (FEP), aztreonam (ATM), meropenem (MEM), amikacin (AMK), gentamicin (GEN), ciprofloxacin (CIP), and trimethoprim/sulfamethoxazole (SXT). Isolates resistant to three or more antibiotic classes were considered multidrug-resistant organisms (MDRs) [33].

We adopted the Simple Disk diffusion method using modified muller-hinton agar as described by Uwizeyimana et al. to test the susceptibility of all the multidrug-resistant (MDR) gram-negative bacteria (GNB) (32/67) to colistin [34]. Briefly, an isolate suspension equivalent to 0.5 McFarland standard was prepared and plated on modified Mueller–Hinton agar 30% (5.1 g/L) (Oxoid, United Kingdom), allowed to dry for 3–5 min, a 10 mg colistin disk was placed and incubated overnight at 35℃ in 5% CO2. The results were interpreted based on the inhibition zone and compared with Minimum Inhibitory Concentrations (MICs) determined by broth microdilution according to CLSI guidelines. *E. coli* (ATCC 25922) and *P. aeruginosa* (ATCC 27853) were used for quality control.

### Statistical analysis

All analyses were two-sided and conducted using STATA version 16. We tested the data for normality, described continuous data in means and medians, and categorical data in frequencies and presented them in tables and figures. Bivariate analysis was performed using logistic regression where crude odds ratio (COR) were calculated. Variables with p ≤ 0.2 were subjected to a multivariate analysis where adjusted odds ratio (AOR) were calculated and significant variables identified. Level of statistical significance was evaluated at p < 0.05, at 95% Confidence Interval (95% CI). Factors found statistically significant are indicated in bold, (Tables 2 and 3).

## Results

### Socio-demographic and clinical characteristics of COVID-19-positive patients admitted at KNH-IDU

We sampled 120-RT-qPCR confirmed COVID-19-positive patients in this study. The majority of the patients were: 60 years and above (43, 35.8%), female (69, 57.5%), married (66.7, 80%), not vaccinated against SARS-CoV-2 (98, 81.7%), mainly presenting with difficulties in breathing (DIB) (60, 50%), admitted to the critical care unit (CCU) (35, 29.2%), discharged (82, 68.3%) after a median length of stay (6–10 days) (48, 40%), and had other co-morbidities (94, 78.3%), Table 1.

**Table 1:** Sociodemographic and clinical characteristics of COVID-19-positive patients admitted at KNH-IDU

Attributes	Frequency (N = 120)	Percent (%)
Age (years)		
Median (IQR)	49 (32–65)	
≤ 24	18	15.0
25–44	39	32.5
45–59	20	16.7
≥ 60	43	35.8
Gender		
Male	51	42.5
Female	69	57.5
Admission site		
IDU isolation ward	85	70.8
IDU-CCU	35	29.2
Marital status		
Single	40	33.3
Married	80	66.7
Clinical presentation		
Cough	50	41.7
Fever	32	26.7
Chest pain	14	11.7
Nausea	25	20.8
Pneumonia	33	27.5
Vomiting	15	12.5
DIB	60	50.0
Others	12	10.0
Presence of comorbidities		
Yes	94	78.3
No	26	21.7
Comorbidities		
HIV/AIDS	6	6.4
Cancer	16	17
Kidney disease	15	16
Diabetes	14	14.9
Hypertension	11	11.7
Haematological disorders	7	7.4
Liver disease	1	1.1
Vaccinated with SARS-CoV-2 vaccine		
Yes	22	18.3
No	98	81.7
Hospitalization Outcome		
Discharged	82	68.3
Dead	38	31.7
Length of hospital stay (days)		
Median (IQR)	9 (5–12)	
Short stay (0–5 days)	34	28.3
Medium stay (6–10 days)	48	40.0
Long stay (≥ 10 days)	38	31.7

*IQR* Interquartile Range, *IDU* Infectious Disease Unit, *IDU-CCU* Infectious Disease Unit-Critical Care Unit, *DIB* Difficult in Breathing, *HIV/AIDS* Human Immunodeficiency Virus/Acquired Immunodeficiency Syndrome, *COVID-19* Coronavirus disease-2019

### Bacterial infections and their etiologies among COVID-19-positive patients admitted at KNH-IDU

**Fig. 1:**
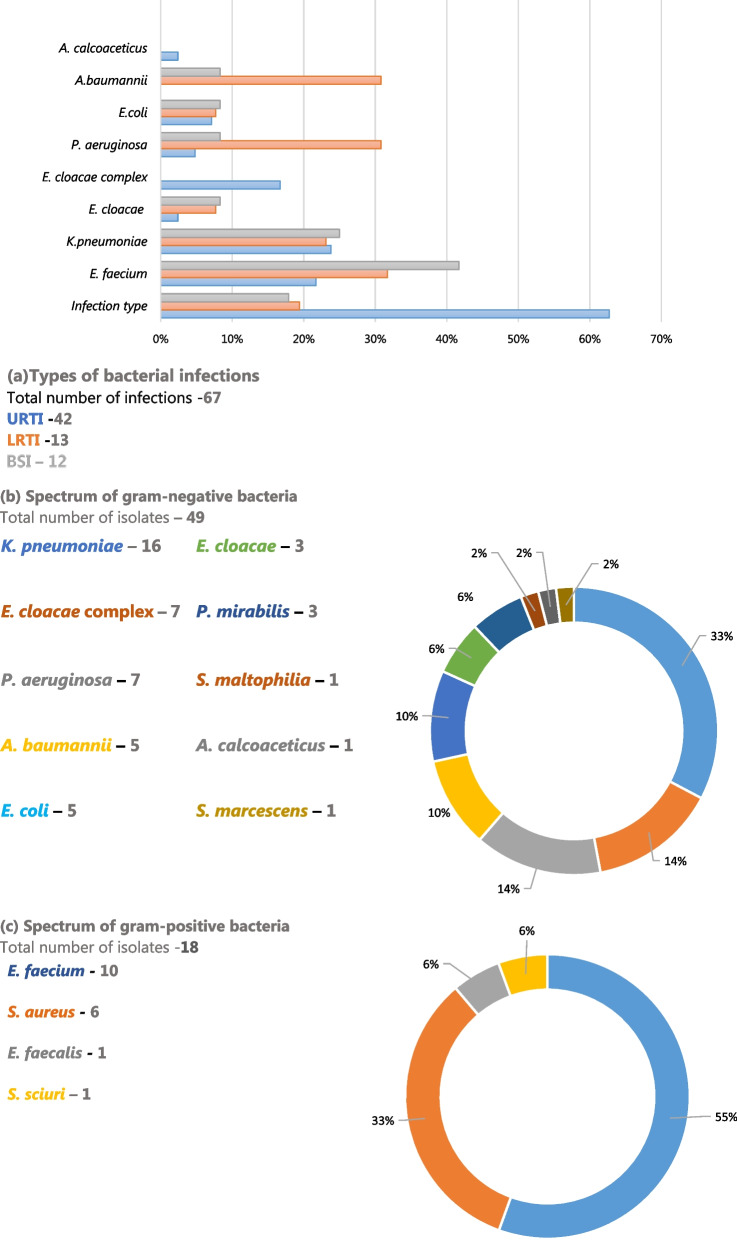
Bacterial infections and their etiologies in COVID-19-positive patients admitted at KNH-IDU. *COVID-19* Coronavirus Disease-2019, *URTI* Upper Respiratory Tract Infections, *LRTIs* Lower Respiratory Tract Infections, *BSI* Blood Stream Infections

The prevalence of bacterial infections among COVID-19 patients was 44.2% (53/120), predominated by upper respiratory tract infections (URTI) (62.7%, 42/67). *Pseudomonas aeruginosa* (30.8%, 4/13) and *Acinetobacter baumannii* (30.8%, 4/13) were the dominant cause of LRTIs, whereas *Klebsiella pneumoniae* was the most prevalent cause of GNB-associated bacteremia (42.9%, 3/7) and upper respiratory tract infections (URTI) (23.8%, 10/42). Overall, *E. faecium* (41.7%, 5/12) was the most common cause of bacteremia, Fig. 1a.

Gram-negative bacteria (GNB) were the most dominant pathogens (73.1%, 49/67), with *Klebsiella pneumoniae* (32.7%, 16/49) and *Serratia marcescens* (2%, 1/49) as the most prevalent and the least common GNB, respectively, Fig. 1b. Among the gram-positive bacteria (GPB) isolates, *Enterococcus faecium* (55%, 10/18) was the most predominant, while the least prevalent were *Enterococcus faecalis* and *Staphylococcus sciuri, *each accounting for 5.6% (1/18) of the isolates, Fig. 1c.

### Factors associated with bacterial infection in COVID-19 patients admitted at KNH-IDU

The male patients were about three times more likely to acquire bacterial infection than their female counterparts (AOR = 2.61, 95% CI: 1.2–5.65, *p* = 0.015), and no other COVID-19 patients' sociodemographic and clinical characteristics were associated with the occurrence of bacterial infections (p > 0.05), Table 2.

**Table 2:** Association between social demographics and bacterial infection in Covid-19 patients admitted at KNH-IDU

Variable	Bacterial infection		COR (95% CI)	p-value	AOR (95% CI)	p-value
	Yes n (%)	No n (%)				
Age						
≤ 24 days	6 (11.3)	12 (17.9)	1.45 (0.56–3.75)	0.211	2.15 (0.54–8.6)	0.281
25–44 years	13 (24.5)	19 (28.4)	2.21 (0.69–7.05)	0.18	1.37 (0.49–3.81)	0.545
45–59 years	13 (24.5)	17 (25.4)	1.62 (0.63–4.13)	0.317	1.45 (0.53–4.01)	0.470
≥60 years	21 (39.6)	19 (28.4)	Ref.		Ref.	
Gender						
Male	30 (56.6)	22 (32.8)	2.67 (1.27–5.6)	0.010**	2.61 (1.2–5.65)	0.015**
Female	23 (43.4)	45 (67.2)	Ref.		Ref.	
Marital status						
Single	13 (24.5)	25 (37.3)	0.56 (0.25–1.25)	0.168	0.7 (0.28–1.79)	0.457
Married	40(75.5)	42 (62.7)	Ref.		Ref.	
Admission site						
IDU isolation ward	40 (75.5)	46 (68.7)	1.41 (0.62–3.16)	0.541	–	–
IDU-CCU	13 (24.5)	21 (31.3)	Ref.			
Presence of comorbidity						
Yes	39 (73.6)	51 (76.1)	0.87 (0.38–2.0)	0.833	–	–
No	14 (26.4)	16 (23.9)	Ref.			
Vaccinated with SARS-CoV-2 vaccine						
Yes	14 (26.4)	14 (20.9)	1.36 (0.58–3.17)	0.519	–	–
No	39 (73.6)	53 (79.1)	Ref.			
Hospitalization outcome						
Discharged	37 (69.8)	45 (67.2)	1.13 (0.52–2.46)	0.757	–	–
Dead	16 (30.2)	22 (32.8)	Ref.			
LOS (days)						
Short stay (0–5)	15 (28.3)	20 (29.9)	0.46 (0.19–1.1)	0.082	0.58 (0.21–1.61)	0.296
Medium stay (6–10)	25 (47.2)	22 (32.8)	0.69 (0.27–1.79)	0.449	0.39 (0.15–1.02)	0.056
Long stay (> 10)	13 (24.5)	25 (37.3)	Ref.		Ref.	

Factors found statistically significant are indicated in bold

*IDU* Infectious Disease Unit, *IDU-CCU* Infectious Disease Unit-Critical Care Unit, *COR* crude odds ratio, *AOR* adjusted odds ratio, **Statistically significant, *Ref* Reference, *CI* Confidence Interval, *LOS* Length of Stay

### Hospitalization outcomes of COVID-19 patients admitted to KNH-IDU

The COVID-19 patients likely to have a positive hospitalization outcome (discharged alive) were those: aged between 25 to 44 years (AOR = 0.13, 95% CI: 0.02–0.6, p = 0.009), vaccinated with the SARS-CoV-2 vaccine (AOR = 0.2, 95% CI: 0.05–0.83, p = 0.027) and admitted to the IDU ward (AOR = 3.27, 95% CI: 1.08–6.89, p = 0.031) for a short length of stay (0 -5 days) (AOR = 14.28, 95% CI:3.25–62.76, p < 0.001), Table 3.

**Table 3:** Association between patient characteristics and hospitalization outcome among COVID-19 patients admitted at KNH-IDU

Variable	Hospitalization outcomes		OR (95% CI)	p-value	AOR (95% CI)	p-value
	Discharged n (%)	Died n (%)				
Age (years)						
≤ 24	11 (13.4)	7 (18.4)	0.34 (0.11–1.01)	0.052	0.39 (0.08–1.85)	0.236
25–44	24 (29.3)	8 (21.1)	0.86 (0.28–2.68)	0.796	0.13 (0.02–0.60)	0.009**
45–59	24 (29.3)	6 (15.8)	0.45 (0.16–1.25)	0.125	0.31 (0.08–1.22)	0.093
≥ 60	23 (28)	17 (44.7)	Ref.		Ref.	
Gender						
Male	34 (41.5)	18 (47.4)	0.79 (0.36–1.71)	0.559	–	–
Female	48 (58.5)	20 (52.6)	Ref.			
Marital status						
Single	25 (30.5)	13 (34.2)	0.84 (0.37–1.91)	0.679	–	–
Married	57 (69.5)	25 (65.8)	Ref.			
Admission site						
IDU ward	68 (82.9)	18 (47.4)	5.4(2.29–12.73)	< 0.001**	3.27(1.08–6.89)	0.031**
IDU-CCU	14 (17.1)	20 (52.6)	Ref.		Ref.	
Presence of comorbidity						
Yes	58 (70.7)	32 (84.2)	0.45 (0.17–1.22)	0.173	1.62 (0.31–4.94)	0.061
No	24 (29.3)	6 (15.8)	Ref.		Ref.	
Vaccinated with SARS-CoV-2 vaccine						
Yes	22 (26.8)	6 (15.8)	2.0 (0.72–5.31)	0.247	0.20 (0.05–0.83)	0.027**
No	60 (73.2)	32 (84.2)	Ref.		Ref.	
LOS (days)						
Short (0–5)	11 (13.4)	24 (63.2)	1.09 (0.34–3.48)	0.879	0.66 (0.17–2.61)	0.556
Medium (6–10)	39 (47.6)	8 (21.1)	11.64 (3.77–35.91)	< 0.001**	14.28 (3.25–62.76)	< 0.001**
Long (> 10)	32 (39)	6 (15.8)	Ref		Ref	

Factors found statistically significant are indicated in bold

*IDU* Infectious Disease Unit, *IDU-CCU* Infectious Disease Unit-Critical Care Unit, *cOD* Crude Odds Ratio, *AOR* Adjusted Odds Ratio, *LOS* Length of Stay, **Statistically significant

### Antimicrobial resistance profiles of bacteria isolated from COVID-19 patients admitted at KNH-IDU

**Fig. 2:**
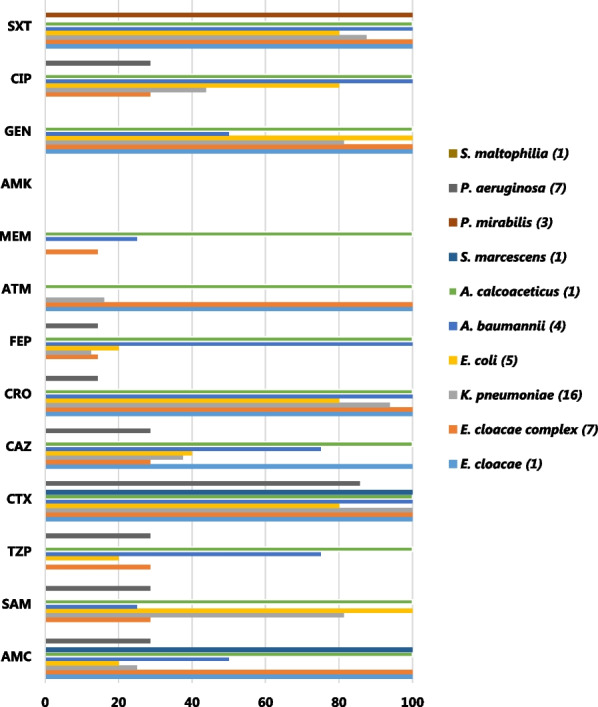
AMR profiles for gram-negative bacteria in COVID-19 patients to IDU at KNH. *AMR* Antimicrobial resistant, *AMC* amoxicillin/clavulanate, *SAM* ampicillin/sulbactam, *TZP* piperacillin/tazobactam, *CTX* cefotaxime, *CAZ* ceftazidime, *CRO* ceftriaxone, *FEP* cefepime, *ATM* aztreonam, *MEM* meropenem, *AMK* amikacin, *GEN* gentamicin, *CIP* ciprofloxacin, *SXT* trimethoprim/sulfamethoxazole

All the GNB isolates were susceptible to amikacin (AMK) but nonsusceptible to gentamicin (GEN), whereas GPB were not resistant to tigecycline (TGC), glycopeptides vancomycin (VAN), teicoplanin (TEC), and oxazolidinones (LZD). *Acinetobacter baumannii* and *Enterobacter *species were non-susceptible to beta-lactamase inhibitor-containing amoxicillin/clavulanate (AMC), Fig. 2.

*Acinetobacter calcoaceticus* was resistant to all β-lactamase inhibitor-containing antibiotics tested, including amoxicillin/clavulanate (AMC), ampicillin-sulbactam (SAM) and piperacillin/tazobactam (TZP), as well as cephalosporins and carbapenems*.* Among the antibiotics tested, *A. calcoaceticus* was only susceptible to amikacin (AMK) and colistin (COL). *Acinetobacter* species were also resistant to third-generation cephalosporins, (cefotaxime, CTX and ceftriaxone, CRO) as well as the fourth-generation cephalosporins, cefepime (FEP). *Proteus mirabilis* were susceptible to all antibiotics tested, except trimethoprim/sulfamethoxazole (SXT, 100%), whereas *Serratia marcescens* isolates were resistant only to AMC and TZP. *Stenotrophomonas maltophilia* were 100% susceptible to all the antibiotic classes tested*,* Fig. 2.

**Fig. 3:**
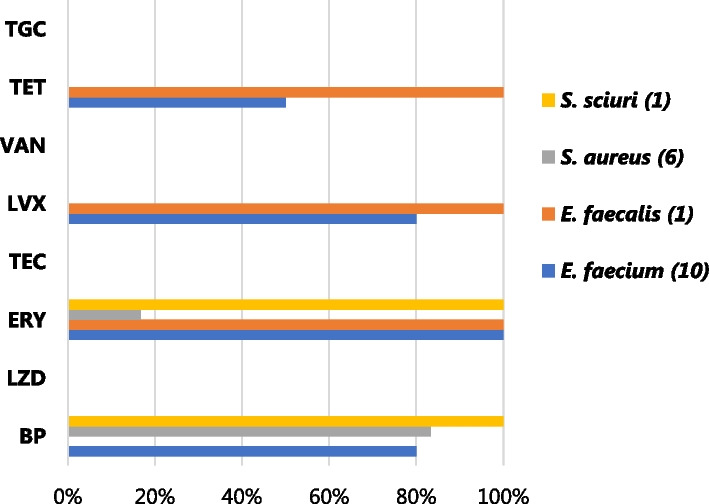
AMR profiles for gram-positive bacteria in COVID-19 patients in KNH-IDU. *AMR* Antimicrobial resistant, *BP* benzylpenicillin, *ERY* erythromycin, *LVX* levofloxacin, *LZD* linezolid, *TEC* teicoplanin, *VAN* vancomycin, *TET* tetracycline, *TGC* tigecycline

All the GPB, except one *S. aureus* isolate (16.7%, 1/6)*, *were 100% resistant to erythromycin. Even though susceptible to the glycopeptides tested (VAN and TEC), *E. faecalis* isolates were 100% resistant to erythromycin, levofloxacin, and tetracycline, Fig. 3.

### MDR phenotypes among the isolates

The majority of bacteria isolates (64.3%, 46/67) were multidrug-resistant (MDR), defined as resistance to three or more classes of antibiotics [35]. Most of the MDR organisms were attributable to GNB (69.6%, 32/46), and all isolates of *Klebsiella pneumonia* (100%, 16/16), *Enterococcus cloacae* complex (100%, 7/7), *Escherichia coli* (100%, 5/5), and *Acinetobacter calcoaceticus* showed multidrug-resistance.

The predominant MDR phenotypes were those observed in *Enterococcus cloacae* (42.9%, 3/7), *Klebsiella pneumonia* (25%, 4/16), and *Escherichia coli* (40%, 2/5) and mostly involved beta-lactamase inhibitors (AMC and SAM), cefotaxime, ceftriaxone, gentamicin, ciprofloxacin, aztreonam and trimethoprim/sulfamethoxazole. Among the GPB, MDR phenotypes were majorly associated with *Enterococcus faecium* and mostly involved benzylpenicillin, erythromycin, levofloxacin, and tetracycline. Notably, erythromycin resistance was present in all GPB-MDR phenotypes, (Table 4). All the MDR GNB, except for *Klebsiella pneumoniae* (31.2%, 5/16) and *Escherichia coli* (40%, 2/5)*,* were susceptible to colistin (data not shown).

**Table 4:** MDR phenotypes among the isolates

Bacteria isolate	MDR phenotype	Frequency (n)	Percentage (%)
Gram negative bacteria			
*Enterococcus cloacae complex *(N = 7)	AMC/TZP/CTX/CAZ/CRO/FEP/ATM/MEM/GEN/CIP/SXT	1	14.3
	AMC/CTX/CRO/ATM/GEN/CIP/SXT	1	14.3
	AMC/CTX/CAZ/CRO/ATM/GEN/SXT	1	14.3
	AMC/TZP/CTX/CRO/ATM/GEN/SXT	1	14.3
	AMC/CTX/CRO/ATM/GEN/SXT	3	42.9
*Enterococcus cloacae *(N = 3)	AMC/CTX/CAZ/CRO/ATM/GEN/SXT	1	33.3
	AMC/TZP/CTX/CAZ/CRO/ATM	1	33.3
*Klebsiella pneumonia *(N = 16)	AMC/SAM/CTX/CAZ/CRO/ATM/GEN/CIP/SXT	1	6.3
	AMC/SAM/CTX/CAZ/CRO/FEB/ATM/GEN/SXT	1	6.3
	SAM/CTX/CAZ/CRO/FEB/ATM/GEN/SXT	1	6.3
	SAM/CTX/CAZ/CRO/ATM/GEN/CIP/SXT	1	6.3
	AMC/SAM/CTX/CAZ/CRO/ATM/CIP	1	6.3
	AMC/SAM/CTX/CRO/ATM/GEN/SXT	1	6.3
	SAM/CTX/CRO/ATM/GEN/CIP/SXT	1	6.3
	SAM/CTX/CAZ/CRO/ATM/GEN/SXT	4	37.5
	CTX/CRO/ATM/GEN/SXT	1	6.3
	SAM/CTX/CRO/ATM/SXT	3	18.8
	SAM/CTX/ATM	1	6.3
*Escherichia coli *(N = 5)	AMC/SAM/TZP/CTX/CAZ/CRO/FEB/GEN/CIP	1	20
	SAM/CTX/CAZ/CRO/GEN/CIP/SXT	1	20
	SAM/CTX/CRO/GEN/CIP/SXT	2	40
	SAM/GEN/SXT	1	20
*Acinetobacter baumannii *(N = 5)	TZP/CTX/CAZ/CRO/FEB/GEN/CIP/SXT	1	20
	TZP/CTX/CAZ/CRO/FEB/MEM/CIP/SXT	1	20
	SAM/TZP/CTX/CAZ/CRO/FEB/GEN/CIP/SXT	1	20
	CTX/CRO/FEB/CIP/SXT	1	20
*Acinetobacter calcoaceticus *(N = 1)	SAM/TZP/CTX/CAZ/CRO/FEB/MEM/AMK/GEN/CIP/SXT	1	100
*Pseudomonas aeruginosa *(N = 7)	CTX/CAZ/CIP	1	14.3
Gram positive bacteria			
*Enterococcus faecium *(N = 10)	BP/ERY/LVX/TET	4	40
	BP/ERY/LVX	4	40
*Enterococcus faecalis *(N = 1)	ERY/LVX/TET	1	100
*Staphylococcus aureus *(N = 6)	BP/OXA/ERY/SXT	1	16.7
Total		46	68.7

*AMC* amoxicillin/clavulanate, *SAM* ampicillin/sulbactam, *TZP* piperacillin/tazobactam, *CTX* cefotaxime, *CAZ* ceftazidime, *CRO* ceftriaxone, FEP- cefepime; ATM aztreonam, *MEM* meropenem, *AMK* amikacin, *GEN* gentamicin, *CIP* ciprofloxacin, *SXT* trimethoprim/sulfamethoxazole, *BP* benzylpenicillin, *ERY* erythromycin, *LVX* levofloxacin, *LZD* linezolid, *TEC* teicoplanin, *VAN* vancomycin, *TET* tetracycline, *TGC* tigecycline

## Discussion

In viral respiratory tract infections, bacterial infections (BIs) contribute to patients’ poor prognosis, increased risk of ICU admission, and mortality ranging from 29 to 55% [14, 16, 36]. Here, we report a 44.2% (53/120) prevalence of BIs among COVID-19 patients admitted to KNH-IDU. Bacterial coinfections in COVID-19 remain controversial. In a multicenter analysis of the clinical microbiology and antimicrobial usage in hospitalized patients in the US with or without COVID-19, Puzniak et al. found a 28% prevalence of co-infections, with 80% as bacterial pathogens [37]. Alshaikh and others, in a systematic review of twenty-two (22) hospital-based studies, reported a 5.62% pooled estimate for the prevalence of bacterial co-infection among adults with RT-PCR confirmed diagnosis of COVID-19 [38]. In our study, clinical presentation suggestive of co-infection informed the sample collection in patients admitted to KNH-IDU, with a potential of higher bacterial isolation as observed here [38, 39]. Though we did not collect samples at the point of admission, previous studies have documented a low prevalence of community-acquired co-infections (≤ 3.5%) among COVID-19 patients [17, 38–41], suggesting that the majority of infections in our study were nosocomial.

The most dominant bacterium causing infections in COVID-19 patients varies widely across the published literature, with *Pseudomonas aeruginosa* and *Escherichia coli* [43], *Staphylococcus aureus* [13, 39] *Streptococcus pneumonia* [43] and *Escherichia coli* [44] as the most common bacterial isolates. In our study, *Klebsiella pneumoniae* (23.9%, 16/67) was the most common bacterial isolate, similar to that recorded by Said and colleagues [45]. Though the hypervirulence mechanisms of *K. pneumonia* are unclear in COVID-19, MDR and hypervirulent pathotypes contribute to the global epidemiology of this pathogen[45]. All *K. pneumoniae* in our study were MDR and 31.2% resistant to colistin, which may explain the predominance among the study population.

Neto and others found genitourinary as the most frequent source of bacterial coinfections in patients with COVID-19 and attributed their finding to higher rates of hypertension, diabetes mellitus, and higher body mass index (BMI) [46]. In our study, the upper respiratory was the predominant source of BIs. Zhu et al. reported the dominance of respiratory pathogens among COVID-2019 cases caused most commonly by *Streptococcus pneumoniae*, followed by *Klebsiella pneumoniae* and *Haemophilus influenzae* [14]*.* However, the bacterial spectrum in our study, except for *Acinetobacter calcoaceticus*, was not the typical upper respiratory tract (URT) colonizers [40, 47], suggesting nosocomial transmission as the possible explanation for the dominance of URI bacterial isolates.

We found that male COVID-19 patients were significantly at high risk of bacterial infection compared to female patients. Saeed and others in the Kingdom of Bahrain [41] documented a similar finding. Women have a stronger immunity to bacterial infections [48]. Coupled with differences in lifestyles such as higher smoking and drinking, adherence to treatment, and attitudes toward the Covid-19 preventive measures, including frequent handwashing [48, 49]. Females' stronger immunity to infections may explain the observed gender-based distribution of BIs in our study.

A growing number of publications show age-related COVID-19 mortality. Ho et al. observed that participants aged 75 and above without other risk factors were four times at risk (95% CI 1.57–9.96, P = 0.004) of death compared with those 65 years and below [50].Zhang and colleagues showed that patients aged > 80 years (*OR* = 1.033 [95% *CI* 1.008–1.059], *p* = 0.01) and male gender (*OR* = 1.585 [95% *CI* 1.301–1.933], *p* < 0.001) were associated with higher odds of death [51]. In our study, the COVID-19 patients likely to have a positive hospitalization outcome were those aged between 25 to 44 years (AOR = 0.13, 95% CI: 0.02–0.6, p = 0.009). Kim et al. observed that the risk of dying increased in COVID-19 patients older than 65 years (OR: 3.08; 95% CI: 1.66–5.71) [52]. Advanced age presents a risk of death in COVID-19 patients due to the likelihood of having other risk factors, such as acute myocardial infarction, acute liver injury, poorer lung function, respiratory failure, hypertension, and acute ischemic stroke[50–52]. Though deaths were not significantly associated with patients' co-morbidities in our study, other factors, including non-SARS-CoV-2 vaccination and prolonged ICU admission, could have contributed to the observed age-dependent mortality. Age dependent defects in T-cell and B-cell function and overproduction of type II cytokines could also lead to a deficiency in the regulation of viral replication and prolonged inflammatory responses, possibly leading to poor outcomes [53].

We found that COVID-19 patients vaccinated against SARS-CoV-2 infection were less likely to die (AOR = 0.2, 95% CI: 0.05–0.83, p = 0.027). Stepanova et al. reported a similar finding, where SARS-CoV-2 vaccination was associated with lower inpatient mortality (OR = 0.47 (0.34–0.65), p < 0.0001) [54]. Patients admitted to the IDU ward (AOR = 3.27, 95% CI: 1.08–6.89, p = 0.031), and those with a short length of stay (0 -5 days) (AOR = 14.28, 95% CI: 3.25–62.76, p < 0.001) in our study, were likely to have a positive outcome. Similarly, Kim et al. observed that the risk of dying increased in COVID-19 patients admitted to the ICU (OR: 6.31; 95% CI: 3.63–10.95) [52]. A prolonged hospital stay increases the risk of bacterial colonization and the development of infection [55] by MDR organisms, thus increasing the risk of death [56].

Increased and indiscriminate consumption of antibiotics during the COVID-19 pandemic is likely to negatively impact AMR, with far-reaching implications on global health and the economy [26, 27, 29]. In our study, all the GNB isolates were susceptible to amikacin (AMK) but resistant to gentamicin (GEN) among other antimicrobial agents. Stefanini et al. showed high bacterial resistance to amikacin and gentamicin in COVID-19 patients [42]. Omar et al. reported 100% and 98% resistance in *Klebsiella pneumoniae* for AMK and GEN, respectively [57]*.* These findings suggest a diminishing clinical value of these aminoglycosides for bacterial coinfections treatment in COVID-19 patients.

Stefanini and others also reported high resistance to aztreonam (monobactam), levofloxacin (third-generation fluoroquinolone), and meropenem (beta-lactam) among bacterial isolates from COVID-19 patients [42]. In our study, *Enterobacter* species and *Acinetobacter baumannii* were non-susceptible to amoxicillin/clavulanate (AMC), a beta-lactamase inhibitor-containing amoxicillin. *Acinetobacter calcoaceticus* was resistant to all β-lactamase inhibitor-containing antibiotics (ampicillin-sulbactam, SAM and piperacillin/tazobactam, TZP), cephalosporins, and carbapenems. *A. calcoaceticus* was only susceptible to amikacin (AMK) and colistin among the tested antibiotics. All the *Acinetobacter* species were resistant to third- (cefotaxime, CTX and ceftriaxone CRO) and fourth-generation cephalosporins (cefepime, FEP). The observed high resistance suggests poor adherence to antibiotic use and infection prevention policies in our study setting and beyond.

In our study, the majority of bacteria isolates (64.3%, 46/67) were multidrug-resistant (MDR), especially in GNB (69.6%, 32/46), with all isolates of *Klebsiella pneumonia* (100%, 16/16), *Enterococcus cloacae* complex (100%, 7/7), *Escherichia coli* (100%, 5/5), and *Acinetobacter calcoaceticus* presenting as MDR organisms. Saeed et al. [41] reported a 65.8% rate of MDR in GNB among patients infected with SARS-CoV-2 in the Kingdom of Bahrain. Polly and others observed an overall 23% increase in MDR infections increased during COVID-19 in an acute care hospital in Brazil [58]. In critically ill COVID-19 patients, MDR bacterial infections increase the length of stay, and their incidences range from 32 to 50% [23]. Invasive mechanical ventilation, steroid therapy, and prolonged ICU stay may play a pivotal role in MDR bacteria emergence and spread [23, 55] suggesting the possible reasons for the observed high MDR infections in our setting.

Weak antimicrobial use policies in developing countries allow improper consumption of the World Health Organization (WHO) Watch and Reserve antibiotics category [30], posing a risk of AMR [26, 27, 29]. High resistance in our study insinuates a near-patient environmental source, indicating compromised hand hygiene besides non-adherence to device-related bundle care practices [30]. A possible additional cause of multidrug resistance is the wide use of biocidal agents for individual and environmental decontamination outside hospital settings [31]. We may have another pandemic of AMR on top of the COVID-19 pandemic, as reported in a recent report that more than 30,000 AMR-associated deaths occurred in Europe alone in the year 2020 [59]. Developing countries are more likely to bear the burden of AMR due to scarcity of potable drinking water, poor sanitation, weak surveillance systems, and poor healthcare systems. We highly recommend culture-based tests for bacterial coinfections in COVID-19 patients to inform judicious prescriptions in the spirit of antibiotic stewardship and systematic continuous surveillance to mitigate AMR.

Despite this being a monocentric study focused on BIs only, sampling unsterile respiratory tract, and due to limited resources to phylogenetically characterize isolates, we present a high burden of bacterial infections in COVID-19 patients with poor hospitalization outcomes and a glimpse at a potential hotspot for MDR infections in our IDU setting. There is therefore, a critical need to rely on culture-based evidence in suspected bacterial co-infections among COVID-19 patients for rationalized antibiotic prescription practices and strictly adhere to hospital infection and prevention policies.

## Conclusion

Our findings highlight a high prevalence of bacterial infections in hospitalized COVID-19 patients, with males more likely to be infected, whilst those in advanced age, not vaccinated with the SARS-CoV-2 vaccine, admitted to the critical care unit, and with prolonged length of hospital stay showing a poor hospitalization outcome. The observed high multidrug-resistant infections are unacceptably high, emphasizing the need to monitor the effectiveness of the existing infection control strategies at KNH-IDU and adherence to antimicrobial stewardship in line with local and global AMR control action plans.

## Data Availability

The datasets used and/or analyzed during the current study are available from the corresponding author on reasonable request.

## Abbreviations

AMR: Antimicrobial resistance
ATCC: American type culture collection
Bis: Bacterial infections
BSI: Blood stream infections
COVID-19: Corona virus disease-2019
GNB: Gram negative bacteria
GPB: Gram positive bacteria
ICU: Intensive care unit
IDU-CCU: Infectious disease unit-critical care unit
KNH-IDU: Kenyatta national hospital-infectious disease unit
LRTI: Lower respiratory tract infection
MDR: Multidrug resistant
NP/OP: Nasopharyngeal/oropharyngeal swab
RT-qPCR: Real Time reverse transcription and quantitative Polymerase Chain Reaction
SARS-CoV-2: Severe Acute Respiratory Syndrome-Corona Virus 2
URTI: Upper respiratory tract infections
